# INTERGENERATIONAL CARE PROVISION AND THE CARE PLANNING OF SANDWICHED AGEING CARERS

**DOI:** 10.1093/geroni/igae098.3860

**Published:** 2024-12-31

**Authors:** Xue Bai, Mengyu Liu

**Affiliations:** The Hong Kong Polytechnic University, Hong Kong, Hong Kong; The Hong Kong Polytechnic University, Hong Kong, Hong Kong

## Abstract

An increasing number of people in their 50s and 60s are sandwiched between surviving parents and children. However, there is a research gap on the relationship between upward and downward care provision of sandwiched ageing carers and the impacts of intergenerational care provision on their care planning. Integrating theories on intergenerational transfer, resource depletion, and proactive coming, this study aims to answer two questions in Hong Kong’s context: (a) How do sandwiched ageing carers provide care across multiple generations? (b) Does intergenerational care provision influence sandwiched ageing carers’ care planning? Using data from the Survey on Sandwiched Ageing Carers in Hong Kong (N = 1500), we find that overall levels of upward and downward care provision of sandwiched ageing carers are positively correlated. However, the high care intensity attenuates such a positive relationship. When the percentage of household income spent for upward and downward care exceeds 12.2%, the association turns to negative. Moreover, the sandwiched ageing carers who provide more upward and downward care are less prepared for their future care. These findings deepen the understanding of intergenerational care provision and care planning experiences among ageing families in Hong Kong and enhance professionals’ and service providers’ awareness of care planning in multi-generational family contexts.

